# Cost-Effectiveness Evaluation of Quadrivalent Human Papilloma Virus Vaccine for HPV-Related Disease in Iran 

**Published:** 2014

**Authors:** Mohsen Khatibi, Hamid Reza Rasekh, Zohreh Shahverdi, Hamid Reza jamshidi

**Affiliations:** a *Department of Pharmacoeconomics and Pharmaceutical Administration, School of Pharmacy, Shahid Beheshti University of Medical Sciences, Tehran, Iran.*; b *Department of Pharmacoeconomics and Pharmaceutical Administration, School of Pharmacy, Shahid Beheshti University of Medical Sciences, Tehran, Iran.*; c *Department of Obstetrics and Gynecology, Shahid Beheshti University of Medical Sciences, Tehran, Iran.*; d *Department of Pharmacology , School of Medicine, Shahid Beheshti University of Medical Sciences, Tehran, Iran. *

**Keywords:** Cost-Effectiveness, HPV vaccine, Iran

## Abstract

Human Papilloma Virus (HPV) vaccine has been added recently to the Iran Drug List. So, decision makers need information beyond that available from RCTs to recommend funding for this vaccination program to add it to the National Immunization program in Iran. Modeling and economic studies have addressed some of those information needs in foreign countries. In order to determine the long term benefit of this vaccine and impact of vaccine program on the future rate of cervical cancer in Iran, we described a model, based on the available economic and health effects of human papilloma virus (HPV), to estimate the cost-effectiveness of HPV vaccination of 15-year-old girls in Iran. Our objective is to estimate the cost-effectiveness of HPV vaccination in Iran against cervical cancer based on available data; incremental cost-effectiveness ratio (ICER) calculations were based on a model comparing a cohort of 15-year-old girls with and without vaccination. We developed a static model based on available data in Iran on the epidemiology of HPV related health outcome. The model compared the cohort of all 15-year old girls alive in the year 2013 with and without vaccination. The cost per QALY, which was found based on our assumption for the vaccination of 15-years old girl to current situation was 439,000,000 Iranian Rial rate (IRR). By considering the key parameters in our sensitivity analysis, value varied from 251,000,000 IRR to 842,000,000 IRR. In conclusion, quadrivalent HPV vaccine (Gardasil) is not cost-effective in Iran based on the base-case parameters value.

## Introduction

The human papilloma virus (HPV) is one of the most common sexually transmitted viruses. Chronic infection with certain subtypes of the HPV is the primary cause of cervical cancer and its precancerous lesions. At least 50% of the adult population is infected with this virus during their lifetime. Despite of screening programs for cervical cancer, it remains the second most common cause of cancer-related death among women worldwide (1, 2). 

Vaccines are essential tools for preventing the diseases. They will protect the vaccinated individual and help to protect the community by reducing the spread of infectious agents (3). 

Gardasil vaccine is part of the national immunization program in the United States, Canada and Australia. It is covered by insurance in Canada and Australia. Gardasil has been registered and used in 124 countries. Moreover, it is part of the national immunization program in 19 countries of the world which means it is administered to all girls aged 11-12 years (4).

In order to determine the long term benefit of this vaccine and impact of vaccine program on the future rate of cervical cancer, many pharmacoeconomists have used mathematical Models. Some models focused on cost-effectiveness of different strategies (5, 6, 7 and 8). 

Human Papilloma Virus (HPV) can cause cervical cancer, genital warts, and some other anogenital cancers (9, 10, 11, 12 and 13). In Iran, HPV types 16 and 18 cause around 72%of cervical cancer, the attribution of HPV 16 and 18 are 56.4% and 15.6% respectively (14). HPV types 6 and 11 cause 90% of cases of genital warts (13, 15). The Estimated percentages of health outcomes attributable to various HPV types showed in Table 1.

**Table1:** Estimated percentages of health outcomes attributable to various HPV types

**Health outcome**	**HPV 6,11**	**HPV 16**	**HPV **18	**Reference**
Cervical Cancer (Iran)	0	56.4%	15.6%	13-
Genital Warts	90%	0	0	( 20- 35- 36-)
Cervical Intraepithelial Neoplasia grade I (CIN I)	6.30%	19.40%	9.20%	33-
Cervical Intraepithelial Neoplasia grade II (CIN II)	0	45.80%	10%	34-
Cervical Intraepithelial Neoplasia grade III (CIN III)	0	45.80%	10%	34-

Numerous studies have been conducted to evaluate its cost-effectiveness by calculating the cost, which is necessary for one quality-adjusted life year (QALY). These include various models such as Markov model, decision model, dynamic model, transmission or a combination one (16).

Studies conducted so far have mostly addressed its impact on cervical cancer and cervical intraepithelial neoplasia (CIN) (17). Few studies have dealt with the cost-effectiveness of Gardasil regarding other malignancies caused by the HPV (18).

The national immunization program of girls prior to sexual activity has shown that it reduces the HPV-related mortality and morbidity and it will be cost-effective (19, 20, 21, 22 and 23). Since HPV infection is asymptomatic, it is growing silently (24).

Vaccination can cause immunity both directly and indirectly through herd immunity (25). Recently a HPV vaccine was added to the Iran Drug List, but it has not been added to the national immunization program till now. Little data exist on the epidemiology of cervical cancer or the prevalence of HPV infection in Iran due to unavailability of national screening program (14).

About 663 new cases of cervical cancer are diagnosed each year in Iran (26), there is no organized population-based cervical screening program, therefore most cervical cancers are diagnosed in late stages, Pap Smear will be done in Iran when they refer to the gynecologist (14). The incidence rate of cervical cancer is low in Iran and many other Muslim countries (27).

The mean cervical cancer ASMR (Age Specific Mortality Rate) for Iran was 1.04 per 100,000 (14). The prevalence of HPV infection was 76% in Iranian cervical cancer patients and 7% among healthy Iranian women (14). The HPV types isolated in Iranian cervical cancer patients includes: HPV 16 (54%), 18 (14%) and 31 (6%) (14).

In Iran, the HPV Prevalence and HPV type is similar to European countries but the rate of Cancer is very low (28).

The US Food and Drug Administration approved a quadrivalent (HPV 6, 11, 16, 18) vaccine (Gardasil, manufactured by Merck and Co.) in June 2006. It was approved for use in girls and women 9-26 years of age (13). The vaccine efficacy will be almost 100% if given to young women before sexual exposures (11, 13 and 29). Other indications have been mentioned in table 2.

**Table 2:** FDA Approval History for Gardasil

**Date**	**approved indications**
Dec, 2010	Gardasil Approved to Prevent Anal Cancer
Oct, 2009	Gardasil Approved for Use in Boys and Young Men
Sep, 2008	Gardasil Approved for Preventing Certain Vulvar and Vaginal Cancers
Jun, 2006	Gardasil Approved for Prevention of Cervical Cancer other HPV-related Diseases

Several cost-effectiveness studies and HTAs (Health Technology Assessments) have been done all over the world for estimation of potential cost-effectiveness of this vaccine; these studies applied a Markov model, a decision model, a dynamic transmission model, or a combination thereof. There was another simplified model that estimated the cost-effectiveness of adding HPV vaccination of 12-year-old girls in the United States (30). This model is very useful for Iran because required data for estimating the cost-effectiveness of Quadrivalent HPV vaccine is available based on this model. Other models are complicated and need many data which are not available in Iran. 

The objective of this study is to estimate the cost-effectiveness of HPV vaccination in Iran against cervical cancer based on available data; incremental cost-effectiveness ratio (ICER) calculations were based on a model comparing a cohort of 15-year-old girls with and without vaccination.

## Experimental

We developed a static model based on available data in Iran on the epidemiology of HPV related health outcome. The model compared the cohort of all 15-year old girls alive in the year 2013 with and without vaccination. 

Similar to the US STUDY MODEL approach, two strategies has been considered; the first one is reference (baseline) strategy including the current situation. As practised in Iran we didn’t consider any national cervical cancer screening. The second one is comparator strategy including the routine quadrivalent (16/18/6/11) HPV vaccination of girls at age 15 with 70% coverage at the year of vaccination.

In other word, the first strategy including the cohort of 15-year-old girl group started from 2013 based on Iranian population without vaccination and another strategy including the same group with vaccination strategy. In both strategies, we used an incidence-based model of the health and economic effects of HPV-related health outcomes including cervical cancer, CIN I. CIN II, CIN III and genital wart. 

We adopted a governmental perspective and included all direct medical costs and benefits regardless of who incurred the costs or received the benefits (31, 32). No other societal costs were included in the analysis. Our study question was “What is the cost per QALY gained by adding vaccination of 15-year-old girls to existing situation (no official screening) in Iran?”

According to a World Bank report published in 2012, the life expectancy of women in Iran is 74 years. We considered a hypothetical population of persons 15-74 years of age. The number of 15-year-old girls was based on 2011 sex-specific population estimates. The number of 16-year-old girls was calculated based on the number of 15-year-olds and the probability of survival from age 15 years to age 16 years. We continued this calculation based on the specific age related mortality for other groups till 74-years in an analogues manner. 

**Table 3:** Parameters and Base Case Value

**Parameters**	**Base case value**
Vaccine efficacy	100%
Protection duration	Lifetime
Time horizon	59-Ys
Vaccine coverage	70%
Euro rate	33,500
Price of Gardasil (Euro)	85.5
Margins	1.460
Vaccine cost	4,181,805
Vials of vaccine needed without considering the booster dose	3
Vaccine cost per series	12,545,415
Financial discount rate (Annual)	3%
Health discount rate (Annual)	3%
Life expectancy (Year)	74

In this cohort we assumed that 3-doses of HPV vaccine will be administered to 15-year-old girls. This cohort will be started in year 15 and will be continued through year 74. Vaccination coverage assumption was 70%. Vaccination efficacy was assumed to be 100% for the HPV 16, 18, 11, and 6 related outcomes (11, 31 and 33). The duration of vaccine protection was assumed to be life-long, but in sensitivity analysis we assumed to administer another booster dose in the year of 10. Based on the proposed price of vaccine in Iran, the cost of full series of quadrivalent HPV vaccine considered 265.5 euro per series.

**Table 4:** Age Specific Incidence Rate (ASIR) per 100,000 in Iran

**Year**	**ASIR(CC)**	**ASIR (CIN I)**	**ASIR(CIN II)**	**ASIR (CIN III)**	**ASIR(FGW)**
10-14 Y	0.07	0	0	0	43
15-19 Y	0	160	80	30	287
20-24 Y	0.22	510	320	130	620
25-29 Y	0.74	140	380	410	394
30-34 Y	1.3	240	140	180	265
35-39 Y	2.75	240	140	180	199
40-44 Y	4.35	120	50	50	139
45-49 Y	6.56	120	50	50	144
50-54 Y	8.91	70	40	10	92
55-59 Y	7.42	70	40	10	86
60-64 Y	6.22	40	10	0	76
65-69 Y	9.42	40	10	0	55
70-74 Y	7.73	20	0	10	40
More than 75	11.7	20	0	10	21
Reference	(25-)	(15-, 36-)	(15-, 36-)	(15-, 36-)	(15-, 36-)

We examined the following HPV-related health outcomes: cervical cancer; CIN grades I, II, and III; and genital warts; the age-specific incidence rates of the HPV-related adverse health outcomes were used to estimate the potential outcomes that could be obtained through their life for both strategies.

Age-specific incidence rates of cervical cancer (ASIR CC) were extrapolated from 2008 population-based cancer registries in Iran (26). Age-specific incidence rates of CIN grades I, II, and III, and female genital warts (FGW) were based on estimates obtained from the literature (16, 37). 

**Table 5:** Treatment cost of HPV adverse health outcomes

**HPV adverse health outcomes**	**Treatment Cost/case (IRR)**
Cervical Cancer:	80,000,000.00
Genital Wart:	2,500,000.00
CIN I:	5,000,000.00
CIN II:	10,000,000.00
CIN III:	30,000,000.00

*Treatment cost of HPV adverse health outcomes*

The cost of care of HPV adverse health outcomes calculated for patients in current clinical practice in Iran in 2013 for each stage of cervical cancer, CIN I, CIN II, CIN III and genital wart. To determine the different kind of cost averted by above mentioned adverse health outcomes, we evaluated the patients files archived in the hospitals and gynecologists private offices. Finally after finding the different kind of direct costs, we referred to the official list of tariffs (38) to calculate the cumulative cost of each HPV adverse health outcomes.

*Costs averted and qalys saved by vaccination*

After calculating the potential HPV adverse health outcomes, we defined the infected population in both strategies. In order to define the cost averted by vaccination, we calculated the cost of infected population via multiplying the infected people in both strategies to the estimated cost of each HPV related disease and then reduced the cost of vaccinated strategy to non-vaccinated strategy. In order to define the QALY averted by vaccination, we calculated the QALY loss of infected population via multiplying the infected people in both strategies to the estimated QALY loss of each HPV related disease and then reduced the QALY loss of vaccinated strategy to non-vaccinated strategy.

To estimate the discounted QALYs lost per case of cervical cancer, CIN I, CIN II, CIN III, and genital warts were based on published estimates of the quality of life without adverse these health outcomes (39) and the estimated reduction in quality of life associated with these HPV-related health outcomes (40, 41, 42, 43), for cervical cancer was assumed to have of six outcomes at diagnosis: 

1- Local lesion lead to survive: QALY loss assumed 0.27 for 4 months and 0.07 for long life till 74 years

2- Local lesion lead to death: QALY loss assumed 0.36 for 3 years and 1 for long life till 74 years

3- Regional metastatic lead to survive: QALY loss assumed 0.37 for 3 years and 0.1 for long life till 74 years

4- Regional metastatic lead to death: QALY loss assumed 0.41 for 3 years and 1 for long life till 74 years

5- Distant metastatic lead to survive: QALY loss assumed 0.45 for 3 years and 0.24 for long life till 74 years

6- Distant metastatic lead to death: QALY loss assumed 0.45 for 3 years and 1 for long life till 74 years. 

Based on expert opinion, we assumed the distributions of cervical cancer stage at diagnosis as follows:

1- Local: 35% and the probability of survival 0.87

2- Regional: 40% and the probability of survival 0.5

3- Distant: 25% and the probability of survival 0.09

For CIN I; we assumed loss in quality of life of 0.03 for 18 months, and no quality loss after this 18 months (42).

For CIN II; we assumed loss in quality of life of 0.07 for 18 months, and no quality loss after these 18 months (42).

For CIN III; we assumed loss in quality of life of 0.07 for 2 years, and no quality loss after these 18 months (42). For genital warts in females, we assumed loss of quality of life and the duration of such loss were assumed to be one of the following four scenarios (42):

1- 0.05 loss for 3 months, with probability of 0.475 

2- 0.1 loss for 6 months, with probability of 0.025 

3- 0.15 loss for 3 months, with probability of 0.475

4- 0.15 loss for 6 months, with probability of 0.025 

*Incremental cost per QALY gained*

Vaccination costs, averted treatment costs and the number of QALYs saved were calculated for each year over a 62-year period, discounted to present value by using an annual discount rate of 3% for both health outcomes and costs (31). 

The incremental cost per QALY gained by using HPV vaccine to existing situation was calculated as (V-A)/Q, where V is the cost of vaccination, A is the averted treatment costs due to vaccination, and Q is the number of QALYs saved due to vaccination (32).

*Sensitivity analysis*

We applied 1-way sensitivity analysis in which we varied one of parameter values while holding other parameters at their base-case values. The parameters we varied included the cost of the vaccine series, the cost per case of all HPV-related health outcomes (±25% of their base-case values); the discount rate (0%, 7.2%); the incidence rates of health outcomes (±25% of their base-case values for CIN 1, CIN 2, CIN 3, and genital warts, and duration of vaccine protection (injection of one booster dose in year 10).

## Results

To calculate the number of woman at each stage, we used the age specific survival rate. Table 6 shows the woman at each stage calculated based on the age specific survival rate.

Life expectancy of women in Iran is 74 years. Therefore, the life expectancy of 15- Years old girls was 59 years. 

**Table 6:** Number of woman at each stage calculated based on the age specific survival rate

**Year**	**1390**	**Year**	**1390**
**10-14 Y**	2,783,047	**45-49 Y**	2,003,143
**15-19 Y**	3,259,607	**50-54 Y**	1,762,295
**20-24 Y**	4,212,922	**55-59 Y**	1,353,485
**25-29 Y**	4,318,020	**60-64 Y**	981,945
**30-34 Y**	3,456,096	**65-69 Y**	700,389
**35-39 Y**	2,720,785	**70-74 Y**	558,821
**40-44 Y**	2,420,370	**More than 75**	895,799

The economic output of interest from the model included total discounted costs and the incremental cost per QALY gained ratio. Both costs and QALYs were discounted at a 3% annual rate (31). We measured the cost-per-QALY ratio as the incremental cost difference between the two strategies divided by the incremental QALY difference between the two strategies. Under base-case parameter values, the estimated cost per QALY gained by adding vaccination of 15-year-old girls to existing cervical cancer screening was around 440,000,000 IRR (Iranian Rial Rate). 

There is much controversy about the choice of discount rates, and whether costs and benefits should be discounted at the same or different rates. In recognition of this, Drummond and Jefferson (44) suggest that sensitivity analysis be done using alternative discount rates, including zero. One reason that zero discount rates have been suggested in both the health and the environmental area is that the benefits are likely to be felt in the future, whereas many costs are incurred now. Considering the zero percent discount rate of cost, the ICER is 414,000,000 IRR. 

For the HPV vaccine, the exact total duration of protection is not known yet, because the current maximum length of clinical trials is around 6 years. Consequently, it could be argued that base-case analysis on the cost-effectiveness of the HPV vaccine should not use durations of protection beyond 6 years, let alone lifelong protection. By considering 10 years protection, the ICER is around 599,000,000 IRR. 

When the cost of treatment for HPV-related complications (Cervical cancer, CIN I, CIN II, CINII, and Genital wart) varied from -25% to +25%, the cost per QALY gained ranged from 447,000,000 IRR to 428,000,000 IRR. When the time horizon varied from 59 to 85 years, the cost per QALY gained ranged from 440,000,000 IRR to 250,000,000 IRR. Changes in the other parameter values such as QALYs that is associated with HPV-related health outcomes also affected the results: when health related outcome varied from -25% to +25%, the cost per QALY gained from 489,000,000 IRR to 387,000,000 IRR. When we excluded the other HPV related complications such as CIN I, CIN II, CIN III and genital wart, the ICER changed to 842,000,000 IRR. In other words, if we consider the prevention of cervical cancer, only the cost per QALY is 842,000,000 IRR.

## Discussion

National decision makers and reimbursement committees prefer to utilize cost utility Analysis. In some countries, decision makers have established ICER thresholds to determine whether a healthcare technology is cost-effective. For example, in the United States, USD50, 000 per QALY gained has been considered as a threshold of cost-effectiveness analysis (45, 46). Similarly in England, the National Institute for Health and Clinical Excellence (NICE) has used ICER of 30,000￡ per QALY gained as a threshold of cost effective for the National Health Service (NHS) (47, 48). 

For developing countries like Iran, World Health Organization (WHO) has recommended a cost-effectiveness threshold indicating that a healthcare technology is cost effective if the ICER is less than three times the GDP (Gross Domestic Production) per capita (49) WHO’s recommendation about threshold of developing countries considers ICER less than triplet of GDP of Iran for 2012 is 5,810 $. Based on WHO recommendation, ICER less than 17,430 USD per QALYs could be considered cost-effective. Since the official exchange rate of USD to IRR is 24,500 in November 2013, the cost-effective threshold in Iran would be around 427,000,000 IRR. In Iran, the availability of data and information for this kind of disease is very limited. Therefore, we used a very simple model in order to estimate the cost-effectiveness of HPV vaccine versus current situation. There is no formal and national screening program for cervical cancer diagnosis in Iran. The cost per QALY, which was found based on our assumption for the vaccination of 15-years old girl to current situation was 439,000,000 IRR. By considering the key parameters in our sensitivity analysis, value varied from 251,000,000 IRR to 842,000,000 IRR. Table 7 shows the summaries of our assumptions and related cost per QALY.

**Table 7:** Summaries of our assumptions and related Cost per QALY

**Assumption **	**Cost of Vaccinated (IRR)**	**QALY loss of Vaccinated**	**Cost of Non Vaccinated**	**QALY loss of Non Vaccinated**	**Cost effectiveness Threshold Iran**	**ICER**	**Is it Cost Effective?**
Base Case	5,527,698,780,568	12,295	1,060,714,746,135	22,468	427,035,000	439,092,468	No
7.2% Financial Discount Rate	5,272,538,157,362	12,295	636,625,601,020	22,468	427,035,000	455,697,686	No
0% Financial Discount Rate	5,902,143,602,544	12,295	1,688,572,121,292	22,468	427,035,000	414,182,699	Yes
Booster Dose	7,157,041,160,945	12,295	1,060,714,746,135	22,468	427,035,000	599,252,425	No
Cost+25%	5,399,764,452,681	2,295	848,571,796,908	22,468	427,035,000	447,369,948	No
Cost-25%	5,687,616,690,428	12,295	1,325,893,432,669	22,468	427,035,000	428,745,618	No
Cervical Cancer Only	5,931,983,028,333	16,687	1,060,714,746,135	22,468	427,035,000	842,587,744	No
Health Related HPV-25	5,403,066,718,428	10,975	855,225,834,442	20,270	427,035,000	489,286,661	No
Health Related HPV+25	5,683,488,858,243	13,944	1,317,575,885,751	25,216	427,035,000	387,351,396	Yes
100 Y Life Expectancy	5,527,698,780,568	19,775	1,060,714,746,135	37,541	427,035,000	251,434,341	Yes

Comparing the cost-effectiveness threshold of Iran to base-case scenario shows that this vaccine is not cost-effective. According to the sensitivity analysis, the most important parameter, which had more and substantial influence, was limiting the indications of Quadrivalent HPV vaccine to cervical cancer only. The time horizon and effectiveness of vaccination during the long life time also had substantial effect on the results. The variation of the cost of treatment had the minimum effect.

Only for the below three scenarios, quadrivalent HPV vaccine is cost-effective in Iran: 

1- If the life expectancy of Iranian women would be 100 years instead of 74 years

2- If the HPV related adverse effect considered 25% more than what we assumed

3- If the financial discount rate assumed zero percent

This study is the first cost-effectiveness study of HPV vaccine and vaccination in Iran and the issue of declining immunity was addressed by assigning a higher cost per vaccination (one dose only) as in the sensitivity analysis to show the cost of a booster. Another advantage of this study is fewer assumptions were needed than more complex Markov and hybrid models did. Since the base-case is established by the incidence rate of different diseases, there is no need to have the following domestic information for modeling: the possible progression from HPV infection to CIN I, CIN II, CIN III, cervical cancer, the probability of HPV infection, the probability of HPV transmission, cervical cancer screening and sexually transmitted disease prevention activities, but the potential benefits of preventing vaginal, anal, vulvar, and oropharyngeal cancers in our estimated cost-effectiveness of HPV vaccine were not considered and the QALY loss related to the initial distress of receiving an abnormal Pap smear result (50) was not considered in this study. Thus, the benefits of HPV vaccination may be underestimated because of reducing the number of abnormal Pap smear results (51, 52). The potential benefits of herd immunity, boys and men vaccination were not considered in this study. As the domestic data for the incidence of CIN I, CIN II, CIN III, and genital warts was not available to us, the international incidence rate has been assumed for mentioned diseases.

In conclusion, quadrivalent HPV vaccine is not cost-effective in Iran based on the base-case parameters values. 
